# The Book World of Medicine and Science

**Published:** 1897-01-09

**Authors:** 


### THE BOOK WORLD OF MEDICINE AND SCIENCE.

A VEST-POCKET MEDICAL DICTIONARY. By ALBERT BUCK, M.D. Price 3s., 529 pages. (London: Ballière, Tindall, and Cox. 1897.)

This is a compact little dictionary neatly bound in morocco, small enough to be easily carried in the waistcoat pocket. The type is very good and clear. It contains such terms and abbreviations as are commonly found in the medical literature of the day, but excludes the names of drugs and of many special anatomical terms. Certain terms taken from the French are included, and their number might with advantage be greater. In general the definitions are short and complete; they are occasionally disappointing; *e.g.*, amylic—containing amyl. There is no definition of this latter body. Ovarian pregnancy—a form of pregnancy, the existence of which is doubtful. This goes near to being an Irishism. A more annoying instance is, perhaps, Pasteurisation—a modified form of sterilisation proposed by Pasteur. The special time and temperature of the process should be given. A few additions of this latter kind, without unduly encumbering the book, would render it more valuable to students.

### YEAR BOOKS.

WHITAKER'S ALMANACK, 1897.

First among the annual publications comes our old friend Whitaker, that constant helper in times of doubt to editors and all who seek for accurate information up to date. There are a few changes and many additions, among the latter being an absolutely perpetual calendar for finding any date from the time of the creation. At least, so says the preface, but we have not as yet been able to work out on what day of the week the world really was created. Of course, in view of new matter inserted every year, old stuff is left out, and the publishers have adopted the very sensible plan of adding an index to former issues from 1869 to 1896.

The Catholic Directory, Ecclesiastical Register, and Almanack, 1897. Price 1s. 6d. (London: Burns and Oates.)

This directory, now in its sixtieth year of issue is published by authority of the Cardinal Archbishop and Bishops of England. It contains an ecclesiastical calendar, followed by an almanack, and much general information, with chapters on the Catholic Hierarchy, on sees, vicariates, &c., in the British empire. The directory itself occupies nearly three hundred pages, and a very useful series of advertisement notices of Catholic colleges, schools, convents, &c., is appended.

The Vegetarian Year Book, 1897. (London: The Vegetarian Federal Union.)

Here we begin with an almanack in which, instead of Saints' Days and great historical events being noted, the dates are filled with matters interesting to vegetarians, and the births and deaths of noted members of that sect. The rest of the book is filled with anecdotes, lives of athletic and long-lived vegetarians, and many extracts from many authors, all of which, the authors hope, will make it "a generally useful hand-book to be used by speakers and others."

